# The clinical value of combined use of MR imaging and multi-slice spiral CT in limb salvage surgery for orthopaedic oncology patients: initial experience in nine patients

**DOI:** 10.2478/v10019-012-0020-4

**Published:** 2012-04-19

**Authors:** Jie Xu, Jun Shen, Yue Ding, Hui-Yong Shen, Zhan-Peng Zeng, Ruo-Fan Ma, Chun-Hai Li, Bertram Barden

**Affiliations:** 1 Department of Orthopaedic Surgery, Sun Yat-Sen Memorial Hospital of Sun Yat-Sen University, 107 Yanjiangxi Road, 510120, Guangzhou, P. R. China; 2 Department of radiology, Sun Yat-Sen Memorial Hospital of Sun Yat-Sen University, 107 Yanjiangxi Road, 510120, Guangzhou, P. R. China; 3 Department of Orthopaedic Surgery, Düren Hospital, Academic Hospital of University of RWTH Aachen, Roonstr. 30, 52351 Düren, Germany

**Keywords:** limb salvage surgery, magnetic resonance imaging (MRI), computed tomography (CT)

## Abstract

**Background:**

The purpose of this prospective study was to evaluate the value of the combined use of MR imaging and multi-slice spiral CT for limb salvage surgery in orthopaedic oncology patients.

**Patients and methods:**

Nine consecutive patients with lower/upper limb malignant bone tumours (7 osteosarcomas and 2 chondrosarcomas) were treated with limb-salvaging procedures. Preoperative planning including determination of the osteotomy plane and diameters of the prosthesis was performed basing on the preoperative CT and MR images. The histopathology was performed as golden diagnostic criteria to evaluate the accuracy of CT and MR-based determination for tumour’s boundary.

**Results:**

The tumour extension measured on MRI was consistent with the actual extension (P>0.05, paired Student’s *t* test), while the extension measured on CT imaging was less than the actual extension. The length, offset and alignment of the affected limb were reconstructed accurately after the operation. An excellent functional outcome was achieved in all patients.

**Conclusions:**

In the present study, MRI was found to be superior to CT for determining the tumour extension, combined use of MRI and CT measurement provided high precision for the fit of the prosthesis and excellent functional results.

## Introduction

Better adjuvant therapy, improved metal implants, and innovative surgical techniques have led surgeons to consider limb salvage surgery as an alternative treatment for malignant bone tumour other than amputation. Orthopaedic oncology patients have a chance for an active, disease-free life after limb salvage surgery. In the first evidence-based study, Simon *et al.* had reported the benefits of limb-salvaging procedures for bone tumours.^1^ Their multicentre study reported the rates of local recurrence, metastasis and survival in 227 patients with osteosarcoma in the distal femur and suggested that the Kaplan-Meier curves of the patients without recurrence were not statistically different between limb-salvaging surgery and amputation patients during a 5.5-year follow-up. Limb-salvage surgery was considered as safe as an amputation in the management of patients with high-grade osteosarcoma.

The goal of limb-salvaging surgery is to preserve the function of limbs, prevent tumour recurrence, and enable the rapid administration of chemotherapy or radiotherapy.^2^ It can be reached with meticulous technique, detailed operative planning, and the use of endoprosthetic replacements and/or bone grafting. For a successful limb-salvage surgery in high-grade malignant tumour, such as sarcomas, a wide margin is necessary to obtain a local control.^3^–^5^ Since marginal and intralesional margins are related to local recurrence, the reconstruction with limb-salvaging options should be carefully considered. The clinical outcome of the limb-salvage surgery with arthroplasty is closely related to the accuracy of the surgical procedure. To improve the final outcome, one must take into account the length of the osteotomy plane, as well as the alignment of the prosthesis with respect to the mechanical axis in order to keep the balance of the soft tissues. Furthermore, the parameters measured with the 3D imagine must be used during the individual manufacture of implant in order to reconstruct the skeletal structure accurately. Therefore, geometric data (such as length of leg, offset) and morphologic data are required.

Magnetic resonance imaging (MRI) was beneficial for tumour detection and consequently staging of musculoskeletal neoplasia. MRI became an ideal imaging modality for musculoskeletal neoplasia because of superior soft-tissue resolution and multiplanar imaging capabilities and had a significant impact on the ability to appropriately stage lesions and adequately plan for limb-salvage surgery.^6^,^7^ In contrast, multi-slice spiral computed tomography (CT) could provide super three-dimensional morphological delineation of the diseased bone. Theoretically, the complimentary use of these two imaging modalities could give the surgeon a more accurate way to implement preoperative planning than the conventional application of 2D images.

The purpose of this prospective study was to report our initial experience with limb salvage surgery for orthopaedic oncology patients by using both MR imaging and multi-slice spiral CT for preoperative planning.

## Patients and methods

### Patients and preparation

The study protocol has complied with all relevant national regulations and institutional policies and has been approved by the local institutional ethics committee. Informed consent was obtained from all patients before the procedure. Patients with malignant bone tumours of lower/upper limb were enrolled in the study.

Preoperative work-up consisted of history and clinical examination, routine laboratory tests and an aesthetic assessment, plain radiography of the limb, 64-slice spiral CT scan of the limb and chest, Technetium-99m bone scan, and, in all of the cases, MRI of the affected limb. Antibiotics were administrated before the surgery. Biopsy was performed for pathological examination. Chemotherapy was commenced 6 weeks before the surgery in those cases which were diagnosed as osteosarcoma and dedifferentiated chondrosarcoma. Patients were classified according to the Enneking staging system.^8^,^9^

The patients received a detailed narrative of conventional, surgical and amputation options after the limb salvage surgery at their own request. Nine consecutive patients with lower/upper limb malignant tumour of bone (5 women, 4 man, mean age 28.6 years, range: 19–52 years) were treated with limb-salvaging procedures. Lesion size (longitudinal direction), location and histology are summarized in Table 1.

### MR imaging

MR images were performed at a 1.5-T superconductive unit (Gyroscan Intera, Philips Medical Systems, Netherlands) and a synergy surface coil was used. The sequences included transverse, sagittal and coronal turbo spin echo T1- and fat-suppressed T2-weighted images. The parameters of these sequences were TR/TE=400 /20 ms for T1-weighted imaging, TR/TE=3500 /120 ms for T2-weighted imaging and a field of view of 480 mm×480 mm for sagittal imaging and 40 mm×40 mm for transverse imaging and 480 mm×480 mm for coronal imaging with a matrix of 512×512, 4–6 signals acquisition and a slice thickness/gap = 5/0.5 mm. Contrast enhanced sagittal, coronal and transverse T1-weighted imaging were obtained after the intravenous injection of gadopentetate dimeglumine (Magnevist, Schering, Berlin, Germany) with a dosage of 0.2 mmol/kg of body weight.

### Multi-slice spiral CT

CT scan was performed by using a 64-slice spiral CT (Sensation 64, Siemens Medical Systems, Germany). The raw data obtained using an axial collimation of 64×0.6 mm, a pitch of 1.0, a tubular voltage of 120 KV and a tubular current of 360 mAs, were reconstructed into contiguous 1-mm thick slices with an increment of 0.5 mm and a field of view of 376 mm × 376 mm and a matrix of 512 × 512 by using the standard soft tissue and bone algorithm. These thin-slice images were postprocessed by using the techniques of multiplanar reformation (MPR) and volume rendering (VR) to demonstrate the lesion details and perform related measurements.

### Preoperative planning

All preoperative radiographs were evaluated by one radiologist and two consultant orthopaedic surgeons, who were members of the surgical team performing the operations. First, the osteotomy plane was determined separately on CT and MRI. On orthogonal coronal enhanced MR images and CT MPR images, the bulk margin of the tumour in the medullary cavity was defined according to the different signal characteristics or attenuation of the tumour itself and the marrow oedema around the tumour. Then, the maximum distance from the top of the greater trochanter to this tumour margin was measured on orthogonal coronal T1-weigthted MRI images, if the tumour was located in the proximal part of femur. The maximum distance from the knee joint line to the tumour margin was measured for the tumours located in the distal part of femur. The maximum distance was defined as the intramedullary extension of the primary tumour and subsequently was used as a reference for the CT measurement. The osteotomy plane on CT MPR images was defined 30 mm distal from the margin of tumour. This distance was also used to determine the length of the extra medullary part of the prosthesis. After the osteotomy plane had been determined, the detailed shape of the medullary cavity of the preserved part of the femur was assessed using the orthogonal MPR technique for determining the diameter and length of the intra medullary part of prosthesis. Diameters of the medullary cavity at the level of the osteotomy plane and the level of the narrowest plane were measured to determine the diameter of the intra medullary stem of the prosthesis. The length of the intra- medullary stem of the prosthesis should be well matched to the length of medullary cavity of the preserved part of femur, which would be optimal if it had an equal length to the extra medullary part of prosthesis. Finally, the centre axis of the femoral shaft measured on CT was used as a reference. Offset, the distance from the central axis of the femoral shaft and the rotation centre of the femoral head, was the index used to determine the neck length of the prosthesis.

### Surgery

All patients underwent en bloc resection and customized prosthetic reconstruction. An anterolateral incision encircling the biopsy scar was used. Limb-salvage surgery consisted of intentional marginal excision, preserving important structures such as major neurovascular bundles, tendons, and ligaments. The osteotomy plane, 30 mm distal from the primary tumour was confirmed based on MRI for all patients. For patients with lesion in the proximal part of femur/humerus, the customized prosthesis was secured using methylmethacrylate cement after the resection. For patients with the tumour in the distal part of the femur, en bloc resection including the tibial plateau was performed and the customized prosthesis was secured using methylmethacrylate cement in both the tibia and femur after the resection. The extensor mechanism was reconstructed by reattachment of the patellar tendon to the slot on the tibial component. After surgery, functional rehabilitation and neoadjuvant chemotherapy were performed.

### Postoperative measurement

After surgery, the patients were followed with a mean of 13 months (range, 9 to 20 months). The postoperative assessment of prosthesis was performed on plain radiography. The central axis of the femoral or humeral shaft and offset were defined. The vertical distance from the line between the top of bilateral ischial tuberosities to the femoral condylar plane was assessed to evaluate the change of the length of the lower limbs. The change of the length of the upper limbs was not assessed for those humeral tumour cases. Functional evaluation was performed in all patients using the 30-point functional classification system of the Musculoskeletal Tumour Society.^8^

### Statistical analysis

Data were expressed as mean ± SD. All measured values were normally distributed (Kolmogorov-Smirnov test). A paired Student’s *t* test was used to evaluate the differences between preoperative planning and post-operative measurements. Values for *p* < 0.05 were considered statistically significant. The statistical analysis was done with SPSS, version 12.0 (SPSS, Inc.).

## Results

The mean postoperative functional evaluation score was 23.3 ± 2.7 (range, 15–27) according to Enneking’s evaluation. Excellent or good function was achieved in all patients and all patients had preserved stable joint (Table 2). There were no local recurrences, metastases or aseptic loosening determined by bone scan, CT scan, ultrasonic examination and laboratory tests in all patients until the end of the follow-up.

### Accuracy of determination for tumour’s boundary

**FIGURE 1:**
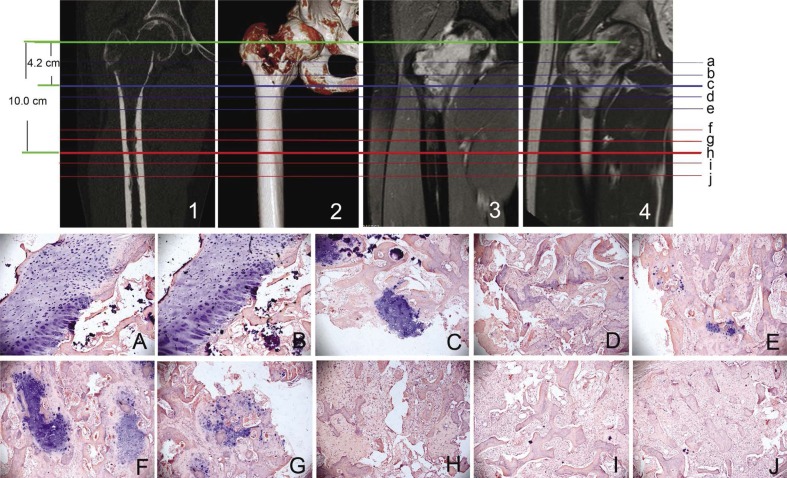
CT and MRI determining of tumour extension. A male, 31-year-old patient with chondrosarcoma in the proximal femur. Coronal MPR image (1), Volume rendering image (2) fat-suppressed coronal T1-weighted image (3) and T1-weighted image (4) showed the tumour in the proximal femur. Distance from the rotation centre of femoral head to the tumour margin in orthogonal coronal CT image and coronal T2-weigthted image was 4.2 10.0 cm respectively. The tumour boundary as determined by MRI and CT were in line c and h respectively. Line a, b, d and e represent the plane 1cm, 2cm around tumour and 1cm, 2 cm to the normal tissue distant from the plane determined by CT. Line f, g, i and j were the plane 1cm, 2cm around tumour and 1cm, 2cm to the normal tissue distant from the plane determined by MRI respectively. A-J are corresponding histologic images (HE, ×200) of line a-j. There was no tumour cells found on the plane h, i, j (Figures H, I, J).

**FIGURE 2:**
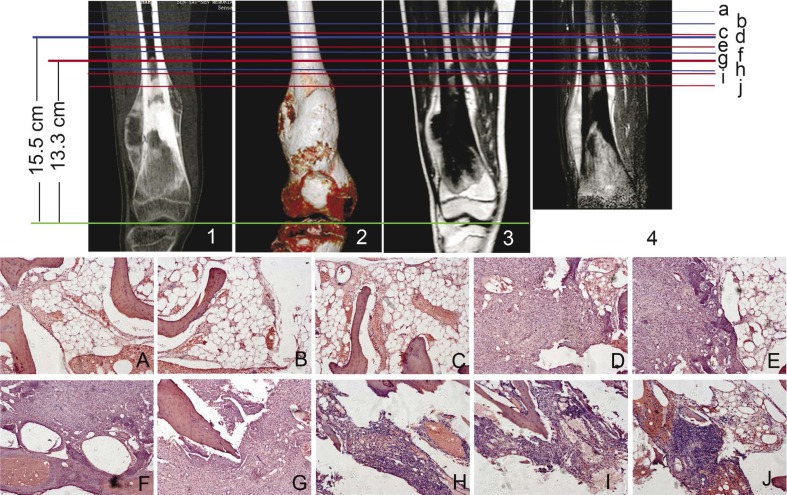
CT and MRI determining of tumour extension. A female, 19-year-old patient with osteosarcoma in the distal femur. Coronal MPR image (1), volume rendering CT image (2), coronal enhanced T1-weighted image (3) and fat-suppressed T2-weighted image (4) showed the tumour in the proximal femur. Distance from the gap of the knee to the tumour margin in orthogonal coronal CT image and on orthogonal coronal T2-weigthed image was respectively 7.2 cm and 8.4 cm. The boundary of tumour as determined by MRI and CT were shown in line c and f respectively. Line i, h, d and b were the plane 1cm, 2cm around tumour and 1cm, 2cm to the normal tissue as determined by CT respectively. Line g, e and a were the plane 1cm, 2cm around tumour and 1cm to the normal tissue as determined by MRI. A–J are corresponding histologic images (HE, ×200) of line a–j. No tumour cells were found on the plane a, b, c (Figures A, B, C).

To determine the accuracy of tumour boundary defined by MRT and CT, the specimens were collected from 1cm, 2cm proximal to the tumour plane and 1cm, 2cm distal as determined by MRI and CT and were examined for histopathology (Figure 1,2).

There was significant difference in tumour extension between MRI and CT measurements (P<0.05). The tumour extension measured on MRI was not statistically different from the actual extension (P>0.05), while the extension measured on CT was less than the actual extension (Table 3).

### Accuracy of reconstruction of the limb length

**FIGURE 3:**
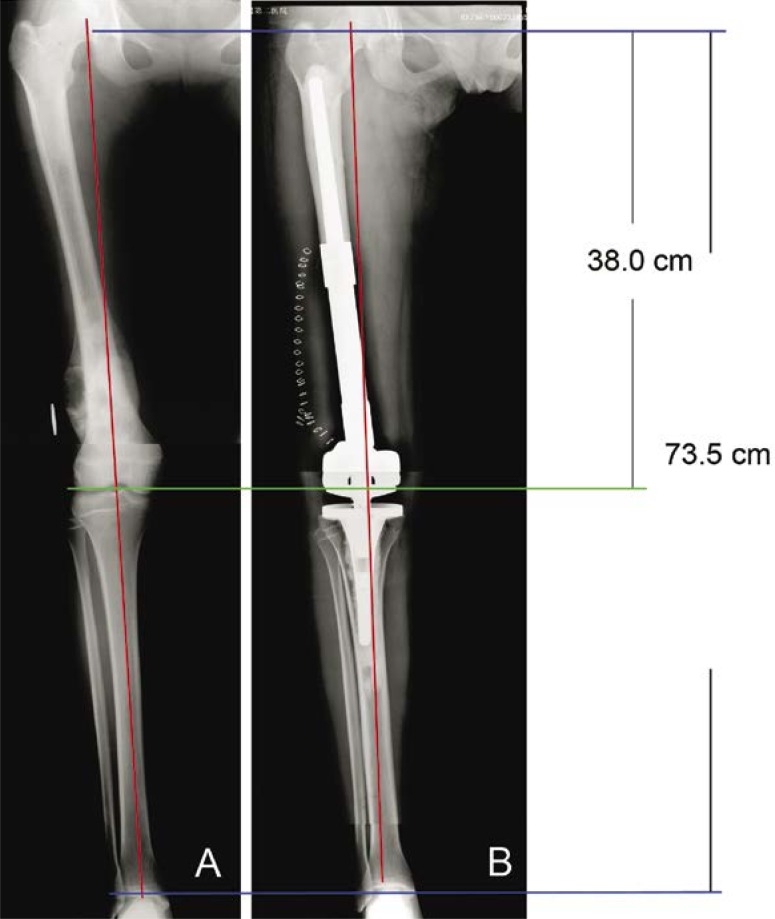
Postoperative assessment of prosthesis. A female, 19-year-old patient with osteosarcoma in the distal femur. Preoperative anterior-posterior plain film (A) and postoperative anterior-posterior plain film (B) reveal that the length and alignment were accurate after reconstruction. The red line showed the alignment of lower limb.

**FIGURE 4:**
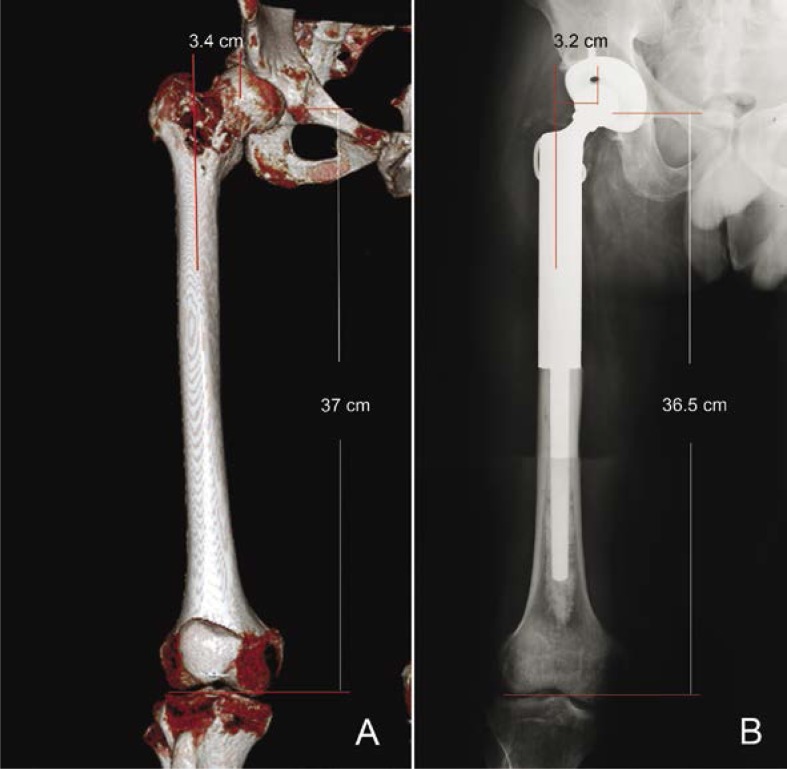
Postoperative assessment of prosthesis. A male, 31-year-old patient with chondrosarcoma in the proximal femur. Preoperative volume rendering images (A) and postoperative anterior-posterior plain film (B) demonstrate that the length and offset were accurately reconstructed.

Before and after operation, there was no significant difference in the length and offset of affected lower limb (Table 4, Figure 3, 4).

## Discussion

### The effect of CT combined with MRI on the determination of invasiveness range of malignant bone tumour

Preoperative imaging plays an important role in determining the stage of bone tumours and then an appropriate choice of therapy for affected patients. An appropriate imaging protocol should always begin with plain radiography. If an aggressive or malignant lesion was suspected, further evaluation with cross-sectional imaging such as CT or MR imaging was needed. CT and MRI are imaging methods, often combined in diagnostic procedures of many oncology tumours.^10^,^11^ CT is useful for a detailed assessment of subtle bony lesions and anatomically complex bones. MRI is particularly useful for determining the tumour extension within medullary compartments and is able to detect tumour involvement of the adjacent muscle compartments, neurovascular structures, and joints. Fat-suppressed T2-weighted imaging proton-density weighted imaging, and contrast-enhanced T1-weighted sequences were frequently used to evaluate neurovascular bundle involvement.^12^–^13^

Currently, MR imaging has become the modality of choice in the local staging of the primary bone tumour.

Many studies have investigated the accuracy of MRI in determining the infiltration range of osteosarcoma. Sundaram *et al.* first reported that MRI would not overestimate the range of osteosarcoma, compared with histology.^14^ Compared with gross and microscopy examination, MRI did not overestimate or underestimate the extent of the tumour, and the false positive and false negative rate were zero. Later, O’Flanagan *et al.* found that MRI could determine the aggression radius of osteosarcoma within the accuracy of 1cm.^15^ For high-grade sarcomas, a wide margin is essential to obtain the local control in order to achieve a successful limb-salvage surgery.^16^–^17^ Meyer *et al.* designed the osteotomy plane according to MRI and found that osteotomy plane could be successfully determined by MRI.^18^ In the present study, the aggression radius of the tumour determined by MRI and the postoperative histological examination was comparable and MRI is superior to CT for determining the tumour extension. Moreover, we found that the result of MRI was slightly larger than the actual extent. The reasons might be that the low signals of peri-tumour oedema was also assigned to the radius of the tumour, resulting in overestimation of tumour size or the preoperative chemotherapy further reduced the aggression radius of the tumour. This result was consistent with the report of O’Flanagan, who found that the aggression radius of the tumour could be evaluated accurately in coronal and sagittal views of T1-weighted images. In contrast it would be overestimated on T2-weighted or fat-suppressed T2-weighted images because of the presence of the peri-tumour oedema. We suggest that MRI was better to demonstrate peri-tumour oedema in comparison to the histological findings. Since this study does not include a long-term follow-up and a large number of patients, a further study is necessary to determine the eventual effect of MRI osteotomy plane on the long-term survival rate.

### The value of three-dimensional CT in the reconstruction of limbs

There is a huge variety in the human skeleton structure as to the size and shape. Therefore, an implant needs to be custom-made to be more suitable for the patient’s bone structure and mechanical requirements. One major challenge is to restore the leg length adequately after the operation.^19^ The leg length discrepancy can affect the joint stability, can cause sciatica and low back pain, and inequable stress on the hip.^20^ Anja *et al* reported that in 1171 cases of total hip replacement most patients with the length of the difference less than 1 cm walked without limp, while 1/4 patients with more than 2cm difference suffered from claudication.^21^ Morrey found that inappropriate eccentricity was one of the factors that could induce dislocation of prosthesis.^22^ Therefore, reducing the eccentricity would increase the risk of dislocation. Dorr *et al.* found that both lack of strength of abductor muscles and impingement of the hip, were the important reasons for dislocation.^23^ Clinically, many factors could lead to hip dislocation. In the presence of the release of soft tissue around the hip and lack of strength of abductor, the decreased offset would significantly increase the incidence of hip impingement syndrome and dislocation, which would increase the instability of the hip joint and may lead to dislocation after slight changes in posture. A smaller offset might lead to excessive loads on prosthesis, and increase the incidence of proximal femoral osteolysis, prosthetic loosening and revision. Theoretically, increasing the offset can reduce the joint reaction force and then may reduce wearing of polyethylene.^24^ Each additional 10 mm of the offset can reduce 10% of the abductor force and 10% less force for the acetabular cup. But if the offset is too large, it can easily lead to malposition of the implant, trochanter projections, local bursitis and pain, and also can affect the transfer of stress and lead to the unequal length of limb.

With the advent of multi-slice spiral CT, the development of an individualized prosthesis became realistic. High accuracy of CT provides a reliable basis for designing the individual prostheses. In this study, the three-dimensional reconstruction of CT images was performed. After the osteotomy plane was initially determined on MRI, the detailed morphological parameters were measured on MPR othorgonal planes. The prosthesis was accordingly designed. This combined use of MRI and CT measurement provided high precision for the fit of the prosthesis and excellent functional results.^25^

## Conclusions

Preoperative evaluation and planning, meticulous surgical technique, and adequate postoperative management are essential for the bone tumour management. In the present study, MRI was found to be superior to CT for determining the tumour extension; the combined use of MRI and CT measurement provided high precision for the fit of the prosthesis and excellent functional results.

## Figures and Tables

**TABLE 1:** Lesion features in six patients

**NO.**	**Primary tumor**	**Sex**	**Age(y)**	**Tumor characteristics**			**Tumor edge disparity between CT and MR(cm)**
				**location**	Size(cm)*	**Size(cm)#**	
1	Osteosarcoma	M	20	Proximal Femur	6.5	6.0	0.5
2	Chondrosarcoma	F	29	Proximal Femur	7.1	5.5	1.6
3	Osteosarcoma	F	21	Distal Femur	15.5	13.3	2.2
4	Chondrosarcoma	M	31	Proximal Femur	10.0	4.2	5.8
5	Osteosarcoma	F	19	Proximal Femur	8.5	7.0	1.5
6	Osteosarcoma	F	52	Distal Femur	9.0	7.6	1.4
7	Osteosarcoma	M	41	Distal Femur	12.9	11.0	1.9
8	Osteosarcoma	F	22	Proximal humerus	14.2	12.3	1.9
9	Osteosarcoma	M	22	Proximal humerus	13.0	11.5	1.5

* measured on MRI;

# measured on CT imaging.

**TABLE 2:** Functional evaluation according to the 30-point functional classification system of the Musculoskeletal Tumour Society

	**Classification(points)**						**Patients Score**
	**5**	**4**	**3**	**2**	**1**	**0**	
**Pain**	None	Intermediate	Modest	Intermediate	Moderate	Severe	4.5±0.7
**Emotional Acceptance**	Enthusiastic	Intermediate	Satisfied	Intermediate	Accepts	Dislikes	4.4±0.5
**Function**	NO Restriction	Intermediate	Recreational Restriction	Intermediate	Partial Disability	Total Disability	4.6±0.5
**Supports**	None	Intermediate	Brace	Intermediate	One cane, One crutch	Two canes, two crutches	3.8±0.5
**Walking Ability**	Unlimited	Intermediate	Limited	Intermediate	Inside only	Unable	4.3±0.7
**Gait**	Normal	Intermediate	Minor Cosmetic problem	Intermediate	Major cosmetic problem. Minor handicap	Major cosmetic problem. Major handicap	3.7±1.0

# The score of postoperative functional evaluation was given as the mean and the standard deviation, which showed that excellent or good function was achieved in all patients.

**TABLE 3:** Accuracy of CT and MRI for determining the tumour extension

	**Tumor margin on CT**	**Tumor margin on MRI**	**Position from tumor margins on CT**				**Position from tumor margins on MRI**			
			**−2cm**	**−1cm**	**1cm**	**2cm**	**−2cm**	**−1cm**	**1cm**	**2cm**
**Positive result determined by histopathologic examination**	9	7	1	9	9	9	0	1	9	9
**Negative result determined by histopathologic examination**	0	2	8	0	0	0	9	7	0	0

The specimens were collected from 1cm, 2cm proximal to the tumours and 1cm, 2cm distal as determined by MRI and CT and were examined for histopathology (which were simplified to 1cm, 2cm, −1cm, −2cm respectively).

# In 9 cases, which were underestimated by CT, positive result of histopathology was determined on 1-cm-point which was distal from CT-determined boundary.

* In 2 cases, which were overestimated by MRI, negative result of histopathology was determined on MR-determined boundary (overestimate).

**TABLE 4:** Preoperative and postoperative measurements of leg length and offset

**No.**	**Contraleral side**		**Preoperative planning**		**Postoperative measurement**		**Disparity between preoperative and postoperative measurement**	

	**Leg length (cm)**	**Offset (cm)**	**Leg length (cm)**	**Offset (cm)**	**Leg length (cm)**	**Offset (cm)**	**Leg length (cm)**	**Offset (cm)**
**1**	39.2	4.1	38.7	4.2	39.4	4.0	0.7	0.2
**2**	36.0	4.0	37.1	4.2	36.6	4.4	0.5	0.2
**3**	38.0	3.6	38.0	3.6	38.0	3.6	0.5	0
**4**	37.3	3.4	37.0	3.4	36.5	3.2	0.5	0.2
**5**	36.5	3.5	36.0	3.6	35.5	4.0	0.5	0.4
**6**	37.5	3.7	37.0	3.7	37.2	3.7	0.2	0
**7**	37.9	3.9	37.7	3.9	37.4	3.9	0.3	0
